# In Times of Crisis

**DOI:** 10.1007/s11569-020-00372-6

**Published:** 2020-07-25

**Authors:** Christopher Coenen

**Affiliations:** Institute for Technology Assessment and Systems Analysis (ITAS), Karlsruhe Institute of Technology (KIT), Karlsruhe, Germany

The Covid-19 crisis has broken the spell. For anyone who does not deliberately close their eyes out of self-interest and is not benighted by conspiracy theories, it has rendered visible the many weaknesses and various strengths of our global system and of national societies.^1^ During the crisis, and perhaps related to it, the largest ever movement to combat racism—a midwife of capitalism and scourge of modernity—emerged in the USA and has spread to many other parts of the world. Even beforehand, developments and events such as the rise of anti-science governments with pro-fascist leanings and religiously fanatic members, increasing international tensions (also between major powers), and the massive bushfires in Australia could be read as signs of a broader global crisis that reveal that our global system is not viable and that humanity senses this.

I am thus very happy that *John Weckert*, our journal’s founding editor, has decided to reflect in our latest issue on both the Covid-19 pandemic and the bushfires, responding to indigenous thinking and thereby contributing to an ongoing discussion about nature. In this discussion, the economist Partha Dasgupta and the UN Environment Programme’s Executive Director Inger Andersen [1] have argued that the Covid-19 crisis shows that we must change our economy and recognise that human wealth depends on nature’s health. In their view, protecting and enhancing our environment must from now on be at the heart of our efforts to achieve economic prosperity. They write that ‘Covid-19 is nature sending us a message’, adding that ‘[i]n fact, it reads like an SOS signal for the human enterprise, bringing into sharp focus the need to live within the planet’s means’ ([1], n.p.). In an interesting caveat against such reasoning, Alan Levinovitz [2] argues that while ‘nature’ may look like a secular term, this framing is fundamentally a religious one; he further points out that religious leaders throughout history have interpreted natural disasters as divine punishments. And he writes ([2], n.p.): ‘The urgency of living in harmony with nature has never been more acute. But we should not make the mistake of believing that living in harmony with nature requires living naturally. After all, the technology required for renewable energy is far less natural than simply burning wood. Refrigeration and freezing prevent spoilage and food waste. Humans are, as the author HG Wells put it, unnatural animals’. In his view, the ‘time has come to abandon our false faith in natural goodness and confront the complexity of what it means to be responsible unnatural animals in a natural world’.

In his article, Weckert argues that as mammals, we are vulnerable, and he urges us to be aware of the consequences of how we treat nature and to be prepared to react to those consequences. Regarding the Earth as our mother, as is widespread in indigenous thinking, might in his opinion prove a useful way of keeping a focus on nature’s importance for human well-being and perhaps survival. For him, it is not about relinquishing agency vis-à-vis nature; we are not victims but rather agents in terms of both the causes and remedies of problems. Ignoring this, he writes, will not promote the well-being of our or any other species.

I will return to our times of crisis at the end of this editorial, but let me first introduce to you the other articles published in *NanoEthics: Studies of New and Emerging Technologies* in 2020. Though the impacts of the crisis unfortunately left me no time to write an editorial for the April issue, you may have already noted that it featured a fascinating special section guest-edited by *Darian Meacham and Miguel Prado Casanova*. For a detailed discussion of its topic—synthetic biology at the intersections of biology, philosophy, art and public engagement with science—it is my pleasure to refer you to their excellent introduction to the special section [3]. The latter includes a wonderful ‘Art-Science Interaction’ piece by *Maria Fannin*, *Katy Connor*, *David Roden* and *Darian Meacham*, a brilliant philosophical discussion of the ethics and ontology of synthetic biology by *Lewis Coyne*, and a rich and beautiful analysis of machine metaphors in discourse on synthetic biology by *Nora Vaage*, an in-depth piece that likewise interacts closely with art. Furthermore, the special section was enriched by *Massimiliano Simons* who painted a highly nuanced picture of notions of engineering and the role they play in synthetic biology and by *Michael Reinsborough* who contributed an engaging discussion of an example of art-science collaboration on synthetic biology and the relevance of such forms of collaboration for public and multi-stakeholder engagement with science and technology. It was a pleasant surprise for me to learn that the support provided by the project SYNENERGENE (2013–2017; see https://www.synenergene.eu/), which I had the honour of coordinating, was deemed useful by participants in this collaboration. Last but by no means least, the special section of our April issue featured an article by *Miguel Prado Casanova*, in which he discusses the theoretical and practical role of ‘noise’ in biological organisation and evolution within the context of synthetic biology; and in a very fine single contribution, *Mareike Smolka* used the example of neurosciences to make an intriguing proposal for how socio-technical integration research (STIR) can contribute to generative critique in interdisciplinary collaborations in the area of responsible research and innovation (RRI).

In our current issue, John Weckert’s contribution is followed by an article by *Sebastian Wäscher*, *Nikola Biller-Andorno* and *Anna Deplazes-Zemp*, in which the authors present the results of a qualitative interview study with senior scientists on the notion and practices of scientific responsibility. Based on their important findings, they argue that two distinct phenomena fall under the notion of scientific responsibility—first, the responsibility grounded in the scientists’ understanding of their roles as scientists and, second, the set of responsibilities in the science system that is not solely connected to the role of the scientists but to many other professional roles—and make practical recommendations against this backdrop. In their article for the August issue, *Halila Faiza Zainal Abidin*, *Kamal Halili Hassan and Zinatul Ashiqin Zainol* analyse and discuss the protection of workers and the regulation of the risks posed by nanomaterials, focusing on soft law approaches. A Malaysian country study that is a worthwhile read in itself, their analysis also acquires a broader relevance when it comes to adopting soft law instruments as regulatory mechanisms for nanotechnology in the context of workplace safety. The August issue also features another contribution on nanosafety aspects, provided by *Neeraj Shandilya*, *Effie Marcoulaki*, *Sven Vercauteren*, *Hilda Witters*, *Eric Johansson Salazar-Sandoval*, *Anna-Kaisa Viitanen*, *Christophe Bressot and Wouter Fransman*. In a first-of-its-kind study, the authors propose guidelines for planning and developing sustainable national centres to deal with the safety of nanomaterials and nanotechnologies, based on a detailed analysis of the current situation, including expert and stakeholder views. The study included 16 national nanosafety centres across Europe and the stakeholder groups of researchers, academics, industry, regulators, civil society and consultants, represented by 44 individuals from 18 EU member states. With its useful figures, the article can also serve as a quick reference tool for practitioners and others wishing to establish a new national nanosafety centre. A Swedish media study on graphene, authored by *Max Boholm*, completes our current issue. It provides a very instructive analysis of the way in which the material is depicted in the media in a national context. Graphene is described, amongst other things, as a super material that is strong, light, thin and highly conductive, usable for various purposes, has societal benefits and may possibly even be revolutioning industry. Negative portrayals are very rare. Notably, Swedish newspapers appear to make only limited reference to graphene as ‘nano’ and only marginal reference to risk. Boholm also discusses his media study results in the broader context of responsible research and innovation and the role of media reporting on science, thereby contributing to a topic that is crucial for the future of RRI.

In our times of crisis, thinking about the future and acting responsibly with respect to future generations have or should become even more relevant. Research fields with a strong societally prospective character thus face specific challenges. My colleagues Martina Baumann, Christoph Schneider, Nora Weinberger, Silvia Woll and I, soon with the support of Julia Hahn, have therefore launched the Rococo initiative (‘Research for orientation and cooperation after the COVID-19 crisis’) with the aim of pooling efforts in such future-oriented fields of study in order to create resources of orientational knowledge for the period after the crisis. During a phase in which almost everything happening in politics and society is understandably dominated by the imperatives of navigating uncharted territory and coping with the pressure for immediate action, these research communities have a particular responsibility to help ensure society does not lose sight of the near and more distant future and to envisage new forms of cooperation. The kind of orientational knowledge needed ranges from the lessons that can be learnt from the crisis about how societies deal with epidemics and pandemics, as well as about healthcare systems more generally, to ideas for the numerous ‘system-relevant’ improvements whose necessity is becoming apparent at this time, and contributions to the comprehensive societal changes that appear desirable in the light of the crisis or could come to be required on account of its socioeconomic consequences. Apart from generating new knowledge, we also intend to gather and harness existing knowledge for those measures that will need to be taken if we are collectively to make our societies more resilient—and that will go far beyond the mere management of epidemics or pandemics. If you are interested in this initiative, please feel to contact me via my institute (contact data: http://www.itas.kit.edu/english/staff_coenen_christopher.php).

Personally and politically, I believe that one key lesson learnt from the Covid-19 crisis is that we should attach greater value to our common humanity, as the crisis makes it appear for example so unnatural that some of us do not even have a home of our own while others live in luxury. The moment the homeless were classified as ‘risk groups’, some hotel rooms—even a few in high-class establishments—were used for them. In the meantime, however, the virus appears to be ‘spreading under the radar in US homeless shelters’ ([4], 128). And researchers ‘working to dampen the toll of COVID-19 in other crowded spaces, such as nursing homes and meat-packing plants, worry that policymakers aren’t concerned enough about outbreaks among marginalized populations’ ([4], 129). Margot Kushel, a researcher–clinician who studies homelessness at the University of California San Francisco, emphasises that she sees it as the role of scientists ‘to raise up these issues and help the public understand how viruses do discriminate, since we live in an inequitable world’ (ibid.).

Noam Scheiber, Nelson D. Schwartz and Tiffany Hsu have summed up another sad lesson learnt during the crisis, namely, how rapidly the liberal-egalitarian make-up of capitalist societies is washed away in stormy times and ‘a kind of pandemic caste system’ can develop: ‘the rich holed up in vacation properties; the middle class marooned at home with restless children; the working class on the front lines of the economy, stretched to the limit by the demands of work and parenting, if there is even work to be had’ ([5], n.p.). The fact that medical staff and other workers deemed to be essential or ‘system-relevant’ (the term used in Germany) are celebrated as heroes is little more than cheap symbolic politics, cheap in the literal sense and akin to the official painting of streets with the ‘Black Lives Matter’ slogan without effecting any real policy change. Though giving higher tips is fine on an individual level, it is rather like giving alms unless it is combined with radically anti-capitalist politics. However, at least the voices of some key workers, as they are usually called in the UK, are now heard more often in even the liberal media (rather than merely in genuinely anti-capitalist publications).

So let us hear from someone who works on the above-cited ‘front lines of the economy’, where orderlies change sheets in hospitals, low-paid staff stack shelves and make deliveries, and homecare providers, cleaners, childcare givers, security guards, postal workers, garbage collectors and others all ‘risk their health to keep America fed, protected and cared for’ ([6], n.p.). Jennifer Medina interviewed Ezzie Dominguez, who is described in the article ‘as a mother of two in Denver, who is a member of the National Domestic Workers Alliance, an association for nannies, caregivers and house cleaners’ (ibid.). Dominguez, the article continues, worked as a nanny and cleaned houses around New York City until, a few weeks ago, the family she was working for told her to stop coming but did not offer to pay her. Her husband, she told the reporter, lost his jobs as a cook and a janitor around the same time and could not collect unemployment benefits because he is an undocumented immigrant. Dominguez considers herself lucky, says the article, because she still has a part-time job at a small nonprofit that provides the family with health insurance, though not enough money for diapers and food for her children. Asked what work she and her colleagues are doing now, Dominguez explained that they are cleaning and sanitising buildings—hospitals for the most part—that are essential to support the public, ‘like on-scene little soldiers, going in, scrubbing down and then disappearing, mostly staying invisible’ (ibid.); they are paid $10 an hour in cash as soon as the work is done. She mentioned that she is in cancer remission and that, when she arrives home, she uses a little room to undress to her underwear, washing everything separately and then jumping in the shower immediately. Dominguez added: ‘Then I kind of hunker on my corner of the bed and stay there, because I am still afraid of touching my husband. I sleep for a couple of hours and then get up to start my job at the nonprofit’ (ibid.). She also reported that when cleaning hospitals, she was not given any kind of protective gear, only two gloves and two little aprons. Asked how the family she had been working for told her they no longer wanted her to come, she responded: ‘I have been working for them for two years. It’s a divorced couple and I am kind of like a secondary mom for the children. They have special needs and the father needs the help at his home. They just said they didn’t want me to come anymore and that they would call me when I can come back. Then they told me they could not contribute anything to me while I am not coming’ (ibid.). And in response to the question of what she wishes people understood about her work right now, she said:

It’s a huge sacrifice. I know I can die if we are exposed, but we are also going to die if we don’t have basic needs. We need the stability of an earned income no matter what. We should not be invisible, but we are all humans, too. We have families and we should be able to have sick leave, so that we can stay home and take care of them. We should not have to choose between working and living. I want Congress to include us, to give us help, too. A crisis is not the time we should say, you need to have documents to have the basic necessities. We should not be excluded from everything. The system is so broken and does not include people like us, even when we are called essential. (ibid.)

History teaches us that capitalism is hard to overcome, and although it becomes more obvious every day that it needs to be overcome or at least extensively transformed if global human civilisation is to survive into the next century, we urgently need to take steps in the right direction within the system. One very simple measure would be to resume collecting significant taxes from the rich and from companies, as was done in the decade after World War II, and to use this tax revenue to make all essential work financially attractive by subsidising it such that all these jobs are put on a par with current upper middle-class incomes. The gentrification of inner cities should be rolled back politically. The fact that metropolitan workers spend hours every day commuting to and from their homes, which in any case are still far too expensive, only to sell bagels and coffee to knowledge workers, capital owners and the non-working rich, to clean the toilets they use or to care for their children, is an absurdity that must end—and not only because it is very costly in ecological terms.

There should also be massive resistance to any attempts to get ‘back to normal’ in agriculture after countries like Australia and Germany temporarily suspended their racist job market regulations for seasonal workers and refugees (who are not usually allowed to work legally in Germany) during the crisis. Scandals sparked by the highly exploitative and unhealthy working conditions faced by migrant workers in the meat industry and other low-income sectors should prompt swift action to address this deplorable state of affairs.

Of course, much of this sounds highly unrealistic given how little organised solidarity the middle classes have displayed—at least in more recent times—towards the lower and working classes; however, in light of what has happened since the 2007/2008 financial crisis, it is not doom-mongering to predict that pressure from the fundamentalist right and those of an openly fascist persuasion will see stable liberal democracies become few and far between if the costs of the crisis are to be borne yet again by the middle and working classes and there is no strong progressive alliance to resist this. This in turn would massively weaken science on account of the anti-science—and indeed anti-reason—agenda of these political forces that Paul Krugman has criticised for the ‘general prevalence of zombie ideas’ in their philosophies, ‘ideas that have been proved wrong by overwhelming evidence and should be dead, but somehow keep shambling along, eating people’s brains’ ([7], n.p.).

What good advice could be given to researchers in this time of multiple global crises? As Petar Jandrić, a researcher in postdigital studies,^2^ points out, the ‘humanities and social sciences are already making significant contributions in areas such as informing citizens, prevention of panic, big data analysis, open science, and others’ ([8], 236). I also agree wholeheartedly with many of his other remarks, namely, that we ‘should not take our home isolations and quarantines as unexpected vacations’ or opportunities ‘to catch up with old projects’ but instead ‘look into the strengths of our disciplinary knowledges and research methods to try and create opportunities to contribute to humanity’s collective struggle against the Covid-19 pandemic and point towards more sustainable futures’ ([8], 237). As Jandrić adds, some of ‘our current insights will be hasted, and will serve as mere first-hand testimonies for later (and more balanced) research’, and in the current ‘infodemic’, most will ‘probably simply remain overlooked and unread’ (ibid.); I can also relate to what he means when he writes:

Wearing my academic researcher hat, I am not ashamed of naivety of this paper—it honestly represents my current thoughts and feelings […]. These thoughts are likely to be overridden by new developments, but they will nevertheless serve as a testimony of this historical moment. Wearing my academic editor hat, I am not afraid of publishing papers that might be proven wrong or even retracted – messy and unpredictable postdigital challenges pertaining to viral modernity require messy and unpredictable attempts at answering. Wearing my Daddy hat, I am admittedly a bit ashamed of withdrawing into the world of research while my son lives through some of the most challenging times in his […] life. Yet beneath all these hats, there is a head; in this head, there is a mind; and in this mind, there is a tiny, persistent voice that whispers: knowledge and solidarity are the key to long-term survival and flourishing of the human race. I invite all postdigital scholars to take this voice seriously, get out of our comfort zones, and explore all imaginable aspects of this large social experiment that the Covid-19 pandemic has lain down in front of us. (ibid.)

Last but not least, I want to express my sincere gratitude—as regards both the April and the new August issue of *NanoEthics*—to Jennyca Parcon and the entire Springer team in charge of our journal for making so many high-quality publications possible in extraordinarily difficult times. Likewise, my special thanks goes to the guest editors of the April special section, Darian Meacham and Miguel Prado Casanova, to all the authors of articles and, also very importantly, to the reviewers of published articles and of papers currently under review or consideration. Let us all hope that the Covid crisis will end well and that we as humanity will be able to learn lessons from it, creating a more just, resilient and sustainable world society.

## Footnotes

1 For one very useful resource on the Covid-19 crisis, see https://the-politics-of-covid-19.com/final-edition/.

2 On the website of the journal edited by Jandrić (https://www.springer.com/journal/42438), the notion of the ‘postdigital’ is discussed as follows: ‘We are increasingly no longer in a world where digital technology and media is separate, virtual, “other” to a “natural” human and social life, and education is often at the forefront of these trends. Journals engaged with technology and education tend to view the research field as concerned with the “effects” of digital media and other technologies on the existing activities of teaching, learning in education, thus continuing to assume a clear division between an authentic educational practice and the imposition of an external, and novel, technology. However, during the past years, we are witnessing a rapid growth in number of academic books and articles dealing (explicitly and implicitly) with education and research in and for the postdigital age’.
